# Directed Evolution of *Mycobacterium tuberculosis* β-Lactamase Reveals Gatekeeper Residue That Regulates Antibiotic Resistance and Catalytic Efficiency

**DOI:** 10.1371/journal.pone.0073123

**Published:** 2013-09-04

**Authors:** Christian Feiler, Adam C. Fisher, Jason T. Boock, Matthew J. Marrichi, Lori Wright, Philipp A. M. Schmidpeter, Wulf Blankenfeldt, Martin Pavelka, Matthew P. DeLisa

**Affiliations:** 1 School of Chemical and Biomolecular Engineering, Cornell University, Ithaca, New York, United States of America; 2 Department of Microbiology and Immunology, University of Rochester School of Medicine and Dentistry, Rochester, New York, United States of America; 3 Laboratorium für Biochemie und Bayreuther Zentrum für Molekulare Biowissenschaften, Universität Bayreuth, Bayreuth, Germany; Institut Pasteur, France

## Abstract

Directed evolution can be a powerful tool for revealing the mutational pathways that lead to more resistant bacterial strains. In this study, we focused on the bacterium *Mycobacterium tuberculosis,* which is resistant to members of the β-lactam class of antibiotics and thus continues to pose a major public health threat. Resistance of this organism is the result of a chromosomally encoded, extended spectrum class A β-lactamase, BlaC, that is constitutively produced. Here, combinatorial enzyme libraries were selected on ampicillin to identify mutations that increased resistance of bacteria to β-lactams. After just a single round of mutagenesis and selection, BlaC mutants were evolved that conferred 5-fold greater antibiotic resistance to cells and enhanced the catalytic efficiency of BlaC by 3-fold compared to the wild-type enzyme. All isolated mutants carried a mutation at position 105 (e.g., I105F) that appears to widen access to the active site by 3.6 Å while also stabilizing the reorganized topology. In light of these findings, we propose that I105 is a ‘gatekeeper’ residue of the active site that regulates substrate hydrolysis by BlaC. Moreover, our results suggest that directed evolution can provide insight into the development of highly drug resistant microorganisms.

## Introduction

Ever since the discovery of penicillin G, β-lactams have emerged as one of the most clinically important classes of antibacterial chemotherapeutics [1]. The mechanism of action for these compounds involves disruption of bacterial cell wall biosynthesis through the irreversible inhibition of transpeptidases that crosslink two peptidoglycan strands. The cross-linked peptidoglycan layer is important for cell wall structural integrity and allows a high internal osmotic pressure to be maintained inside the cell [2]. Despite the wide-ranging clinical utility of β-lactam antibiotics, a significant number of β-lactam-resistant strains have emerged in recent years, compromising our ability to effectively treat bacterial infections. Antibiotic resistance to β-lactams primarily arises through the horizontal transfer of β-lactamase genes contained on plasmids [3], although intrinsic mechanisms not specified by mobile elements (*e.g.*, efflux pumps) are also recognized as key contributors to antibiotic resistance in bacteria [4].

As far back as the 1940s, it was known that β-lactams are ineffective in the treatment of infections caused by *Mycobacterium tuberculosis* [5]. The production of β-lactamase was proposed to be the most significant reason for the intrinsic resistance of *M. tuberculosis* to these antibiotics [6]. In line with this proposal, the genome of *M. tuberculosis* harbors a chromosomal class A (Ambler) β-lactamase encoded by *blaC* (Rv2068c). When *blaC* was deleted from *M. tuberculosis*, the resulting cells exhibited increased susceptibility to β-lactam antibiotics by 8- to 256-fold [7], [8], thereby linking BlaC with the intrinsic resistance to β-lactam chemotherapy. The efficiency with which BlaC thwarts β-lactam chemotherapy stems from its ability to hydrolyze penicillin, cephalosporin, and carbapenem classes of β-lactams [9], [10]. One promising approach to combat the inherent β-lactam resistance of *M. tuberculosis* is to use β-lactam antibiotics in combination with β-lactamase inhibitors such as clavulanate [9], [11].

In this study, we used directed evolution, a technique that can imitate natural evolution, to predict future mutations that might appear in the face of increased exposure to antibiotics. Export of BlaC out of mycobacteria is dependent on the twin-arginine translocation (Tat) pathway [12], which is well known for its ability to transport folded proteins across the cytoplasmic membrane [13]. Only BlaC proteins that possess functional Tat signals can protect mycobacteria from antibiotic challenge [12]. In contrast, truncated versions of BlaC, either lacking an endogenous Tat signal peptide or modified with an export signal specific for the Sec pathway, are unable to confer protection against β-lactam antibiotics [12]. Along similar lines, BlaC has been used as a reporter to identify functional Tat-dependent export signals in *M. tuberculosis* proteins [14], [15]. In these earlier studies, β-lactam resistance was used as a phenotypic indicator of functional BlaC export in mycobacteria by direct selection of drug-resistant colonies on agar containing penicillins such as ampicillin (Amp) or carbenicillin (Carb). Here, we transferred BlaC-mediated β-lactam resistance to fast growing, non-pathogenic *Escherichia coli* cells, thus providing a convenient selection system for rapidly evaluating the effect of genetic or pharmacologic alterations of BlaC activity.

## Results

### Functional Transfer of BlaC-dependent β-lactam Resistance to *E. coli*

**Figure 1:**
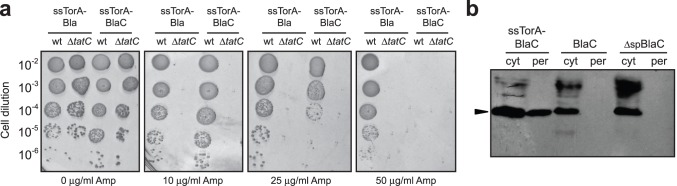
Heterospecific expression of *M. tuberculosis* BlaC in *E. coli*. (a) Serially diluted wt or Δ*tatC* cells expressing ssTorA-Bla or ssTorA-BlaC chimeras were spotted on Amp. (b) Western blot analysis of cytoplasmic (cyt) and periplasmic (per) fractions prepared from wt cells expressing ssTorA-BlaC, full-length BlaC, or BlaC lacking a signal peptide (ΔspBlaC). Arrow indicates BlaC. Samples prepared from an equivalent number of cells were loaded in each lane. Blots were probed with anti-FLAG antibodies.

TEM-1 β-lactamase (Bla) is a close class A homologue to BlaC that confers β-lactam resistance to *E. coli* cells. Since β-lactams target cell-wall biosynthetic enzymes located outside of the cytoplasm, Bla must be exported beyond the cytoplasm to protect the bacterium from the drug. For this reason, when fused to another protein, Bla can be used as a reporter of protein export and folding in *E. coli* [16]–[20]. Here, we attempted to establish β-lactam resistance in *E. coli* cells using *M. tuberculosis* BlaC expression. BlaC is a 307-amino-acid protein that bears an N-terminal Tat signal peptide and is natively exported by the Tat pathway in mycobacteria [12]. For expression in *E. coli*, we swapped the native Tat signal (amino acids 1–30 of precursor BlaC) with an *E. coli* Tat export signal derived from trimethylamine *N-*oxide reductase (ssTorA), resulting in the ssTorA-BlaC chimera. Wild-type (wt) MC4100 *E. coli* cells expressing ssTorA-BlaC were challenged on increasing antibiotic concentrations and found to be resistant up to 25 µg/ml Amp (Fig. 1a). For comparison, wt cells expressing a previously reported fusion between ssTorA and TEM-1 Bla [16] were also resistant, although to a much higher concentration of Amp (Fig. 1a). As expected for Tat-dependent substrates, these constructs failed to confer Amp resistance to Δ*tatC* cells (Fig. 1a), which lack the essential TatC component of the translocase and are thus blocked for export [21]. Consistent with export studies in mycobacteria [12], BlaC was incompatible with the *E. coli* Sec translocase (Fig. S1a). Specifically, BlaC hybrids carrying Sec- (e.g., ssPhoA, ssMalE) and SRP-dependent (e.g., ssDsbA) export signals were susceptible to Amp. Interestingly, cells expressing full-length BlaC with its native export signal were unable to confer Amp resistance to *E. coli* cells (Fig. S1a), suggesting that the *M. tuberculosis* Tat signal of BlaC is not compatible with the *E. coli* Tat translocase. Since export of β-lactamases to the periplasm is a prerequisite for antibiotic resistance in *E. coli* [22], the above phenotypes are easily explained by inspection of the subcellular distribution of BlaC. Specifically, ssTorA-BlaC appeared prominently in the periplasmic fraction whereas full-length BlaC and the Sec/SRP-targeted BlaC hybrids were all retained exclusively in the cytoplasmic fraction in a manner that was indistinguishable from BlaC lacking an export signal (Fig. 1b and Fig. S1b).

### Mutational Pathways to Enhanced BlaC-mediated β-lactam Resistance

Upon successful transfer of BlaC-dependent β-lactam resistance to *E. coli*, we next sought to probe the mutational pathways to enhanced BlaC-mediated β-lactam resistance using our reconstituted system. Specifically, we employed a directed evolution strategy, which mimics natural evolution on a laboratory timescale and has been used previously to predict the order and combination of mutations in TEM-1 Bla that lead to increased resistance [23], [24]. Specifically, an error-prone library of BlaC (0–4.5 mutations/kb) was cloned downstream of the region encoding ssTorA and under the control of the *lac* promoter. We chose to introduce, on average, two mutations per copy into the BlaC-coding sequence so that all possible single-point mutations were sampled and that a fraction of all possible double- and triple-point mutations were sampled in each experiment. This mutation frequency was chosen because in nature, mutations usually occur one at a time and very rarely in pairs. Hence, mutations that only confer a fitness advantage when introduced simultaneously would be unlikely to arise simultaneously in the same genome. *E. coli* MC4100 cells expressing the BlaC library were selected on 200 µg/mL Amp and ∼50 drug-resistant colonies were recovered. To ensure the phenotype was plasmid-associated, plasmids isolated from the selected clones were retransformed in MC4100 cells and the resistance phenotype of fresh transformants was confirmed. Sequencing of ten randomly chosen positive hits revealed that six were clonally identical, encoding a version of BlaC with only a single amino acid substitution of Phe for Ile at position 105 (I105F) of BlaC [the residues of *M. tuberculosis* BlaC were numbered according to a consensus ABL numbering scheme [25]]. The other four sequenced hits were also clonally identical, each carrying a Thr substitution at position 105 (I105T) along with two additional substitutions: T145dI and V263I. However, since the I105F mutation conferred a stronger antibiotic resistance phenotype to *E. coli* cells, we focused our attention on this clone.

**Figure 2:**
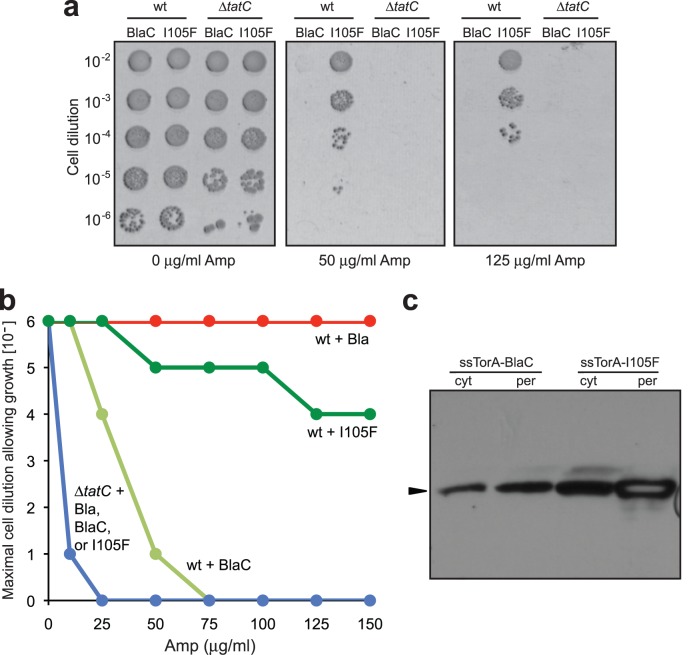
Directed evolution yields enhanced BlaC mutant. (a) Serially diluted wt or Δ*tatC* cells expressing ssTorA-BlaC or ssTorA-BlaC(I105F) chimeras were spotted on Amp. (b) Maximal cell dilution allowing growth as a function of Amp concentration. (c) Western blot analysis of cytoplasmic and periplasmic fractions prepared from wt cells expressing ssTorA-BlaC or ssTorA-BlaC(I105F). Arrow indicates BlaC.

To confirm that BlaC(I105F) conferred increased Amp resistance to cells and retained its dependence on the Tat pathway, we compared the expression of ssTorA-BlaC and ssTorA-BlaC(I105F) in wt and Δ*tatC* strains. Plating of these cells on increasing Amp concentrations revealed that both were exported in a Tat-dependent manner and that BlaC(I105F) conferred significantly increased resistance to Amp (Fig. 2a and b). To further verify Tat pathway exclusivity, we expressed the BlaC(I105F) as a fusion to ssPhoA, ssMalE and ssDsbA. Like its wild-type progenitor, BlaC(I105F) was unable to confer Amp resistance to cells when targeted to the Sec/SRP pathways (Fig. S1c). Subcellular fractionation revealed that both wt BlaC and BlaC(I105F) were localized in the periplasm, although the expression level of BlaC(I105F) in both the cytoplasmic and periplasmic fractions was visibly greater (Fig. 2c). Taken together, these data indicate that the enhanced resistance phenotype conferred by BlaC(I105F) was strictly dependent on a functional Tat pathway and was not due to ‘promiscuous’ rerouting between the Tat and Sec pathways that has been observed for some other proteins [19].

### Activity and Stability of BlaC Mutant are Enhanced

Besides expression level, we hypothesized that the I105F substitution might enhance other properties (e.g., catalytic efficiency, stability) of the enzyme relative to the parental BlaC protein. To test this notion, BlaC and BlaC(I105F) were each cloned in pET-28b with an N-terminal 6x polyhistidine (6x-His) tag followed immediately by a thrombin cleavage site. Following expression in *E. coli* BL21(DE3) cells, each enzyme was purified by Ni-NTA affinity chromatography followed by size exclusion chromatography and thrombin cleavage to remove the 6x-His, as described previously [9], [10] but with one notable difference. Previous reports expressed a truncated version of BlaC that lacked the first 40 amino acids because attempts to express the full-length protein were unsuccessful. In our hands, truncation of the enzyme was not required; we were able to produce large quantities of active, full-length enzymes. The overall yields for full-length BlaC and BlaC(I105F) were 50–100 mg/L of *E. coli* culture and the purity of each was estimated to be >95% according to Coomassie staining (Fig. S2). Initial rate kinetics were used to determine the steady state kinetic parameters for the wt and mutant BlaC enzymes using Amp as substrate. The catalytic efficiency, *k_cat_/K_m_*, observed for wt BlaC was 1.4×107 (Table 1 and Fig. S3) which was in reasonable agreement with previous work [9]. In contrast, the k_cat_/K_m_ value for BlaC(I105F) was nearly 2-fold higher, consistent with the enhanced resistance conferred by this clone above. This improved catalytic efficiency could be ascribed to a measurable increase in k_cat_ and decrease in K_m_ for the mutant compared to the parental enzyme (Table 1 and Fig. S3).

**Table 1:** Kinetic and thermodynamic parameters for wt and mutant BlaC enzymes.

Enzyme	*K_m_* (µM)^a^	*k*_cat_ (min^−1^)^a^	*k*_cat_/*K_m_* (min^−1^ M^−1^)^a^	IC_50_ (µM)^b^	Δ*G* ^25^°^C^ (kJ mol^−1^)^c^	*M* (kJ mol^−1^ M^−1^)^c^	[urea]_M_ (M)^c^
wt BlaC	50±3	720±10	(1.4±0.3) ×10^7^	4.0±0.1	23.4	9.4	2.5
BlaC(I105T/T145dI/V263I)	nd	nd	nd	nd	33.4	11.8	2.8
BlaC(I105F)	32±2	772±14	(2.4±0.1) ×10^7^	8.9±0.6	31.1	11.1	2.8

a Kinetic parameters determined using Amp as substrate (Wang et al., 2006);

b IC_50_ values determined using nitrocefin and inhibitor clavulanate;

c Data analysis according to a two-state-model (Santoro and Bolen, 1988); nd, not determined.

**Figure 3:**
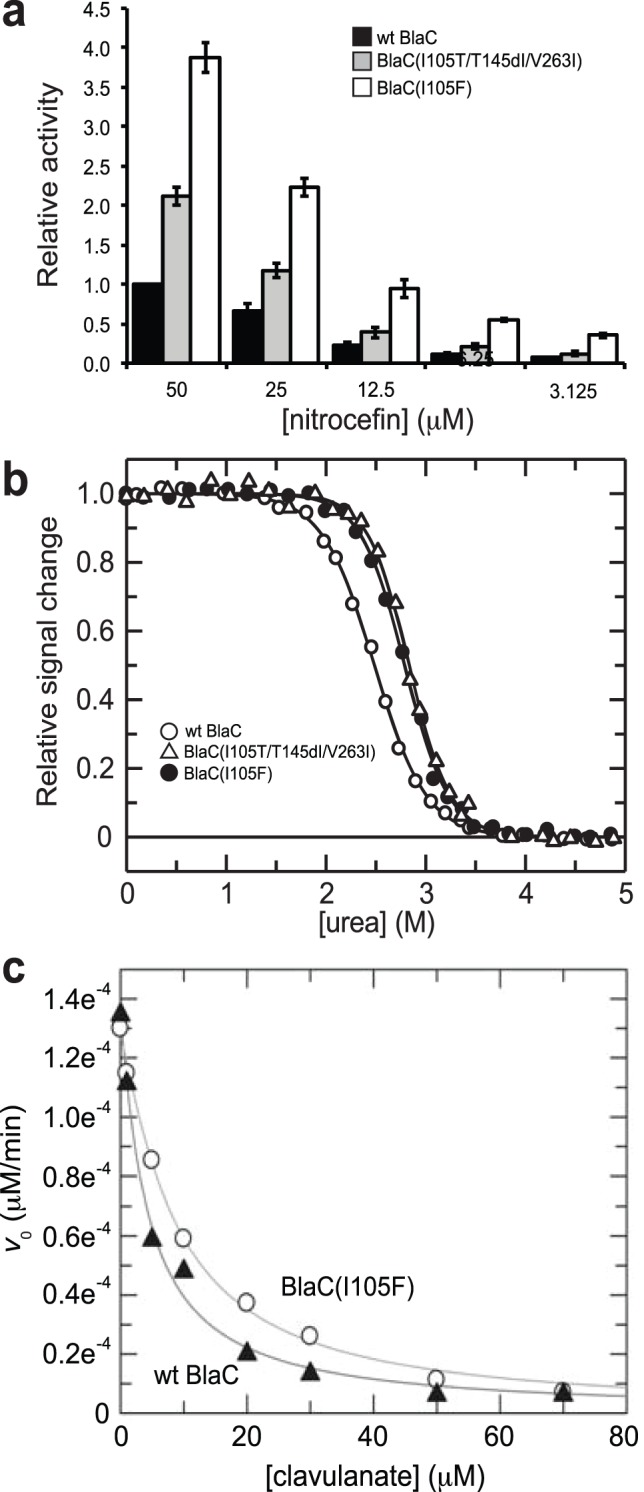
Characterization of BlaC enzymes. (a) Nitrocefin hydrolysis activity in cell lysates. Data was normalized to the activity measured in cells expressing wt BlaC. Data represents the average of 3 replicate experiments and the error bars represent the standard error of the mean. (b) Equilibrium unfolding transitions of purified enzymes. (c) Inhibition of wt BlaC and BlaC(I105F) enzymes by clavulanate measured using nitrocefin as substrate.

Since BlaC exhibits high *k*_cat_ values for many cephalosporins [9], we also used the chromogenic cephalosporin nitrocefin as substrate to evaluate enzyme activity. In the case of BlaC(I105F), nitrocefin hydrolysis was nearly 4-times greater than that measured for the wt enzyme over the same time period (Fig. 3a). For comparison, in the case of the BlaC(I105F/T145dI/V263I) mutant nitrocefin hydrolysis was >2-times greater than the wt enzyme. The *k*_cat_ values for all three enzymes were nearly identical (Table S1 in Tables S1 and Fig. S4) and in close agreement with earlier values [9]. However, *K_m_* values for the two mutants were notably lower than the values measured for the wt enzyme (Table S1 and Fig. S4). As a result, the catalytic efficiency, *k*_cat_/*K_m_*, of the BlaC(I105F/T145dI/V263I) and BlaC(I105F) mutants was increased by up to 1.3 and 3-fold, respectively, compared to the wt enzyme. In addition to enhanced catalysis, the two mutant BlaC enzymes were both thermodynamically more stable than their wt counterpart (Fig. 3b).

### BlaC Mutant is More Active in Presence of Clavulanate

Clavulanate irreversibly inhibits class A β-lactamases including BlaC by rapidly reacting with the enzyme to form hydrolytically stable, inactive forms of the enzyme [9]. To determine whether this inactivation was affected by the I105R mutation, enzymatic activity of purified enzymes was measured with nitrocefin as reporter substrate and inhibition by clavulanate was measured by determination of IC_50_ values. While the inhibition of both enzymes was of similar magnitude, the BlaC(I105F) exhibited an IC_50_ value of 8.9 µM compared to a value of 4 µM for wt BlaC (Fig. 3c and Table 1). The *K_i_* values estimated from these IC_50_ values [26] were 0.29 µM and 0.34 µM for the BlaC(I105F) and wt enzymes, respectively, the latter of which is in close agreement with an earlier report [9]. We next tested *E. coli* strains expressing ssTorA-BlaC or ssTorA-BlaC(I105R) for their susceptibility to Amp in the presence of a fixed concentration of clavulanate. Both enzymes were clearly inhibited by clavulanate as evidenced by the increased Amp sensitivity of cells in the presence of 0.025 and 0.25 µM inhibitor (Fig. S5). While the extent of inhibition was comparable, cells expressing the BlaC(I105R) were still notably resistant to Amp in the presence of clavulanate, reflecting the high catalytic efficiency of the mutant enzyme against Amp.

### BlaC Mutant Increases Antibiotic Resistance of *M. smegmatis*

To evaluate the resistance imparted by BlaC(I105F) in mycobacteria, plasmids carrying either wt BlaC or BlaC(I105F) were transformed in *M. smegmatis* strain PM965, which lacks the *blaS1* gene encoding the primary β-lactam resistance protein in this organism [7]. The *blaS1* deletion in PM965 removes the major β-lactamase enzyme of *M. smegmatis* and allows us to test the drug susceptibilities of this strain expressing the wt or mutant BlaC enzyme. Consistent with the antibiotic resistance phenotype seen above using *E. coli*, *M. smegmatis* Δ*blaS1* cells expressing BlaC(I105F) were significantly less susceptible to Amp as determined by disc diffusion assays (Table S2 in Tables S1). At the highest concentrations of clavulanate tested, the wt and BlaC(I105F) conferred similar Amp resistance to Δ*blaS1* cells and thus were comparably inhibited by clavulanate. However, at lower clavulanate concentrations, cells expressing BlaC(I105F) were less susceptible to Amp (Table S2in Tables S1), reflecting the high catalytic efficiency and/or the modest clavulanate resistance of the mutant enzyme.

#### Structural basis for enhanced catalytic efficiency of mutant BlaC

To understand how the I105F mutation enhanced the catalytic efficiency of BlaC, we looked for clues from the enzyme’s structure. Given that earlier BlaC crystal structures were for a truncated version of the enzyme that lacked the first 40 amino acids [9], [10], we decided to crystallize the full-length version of the enzyme. Crystals were present in the P2_1_ space group, and diffraction data to 2.8 Å resolution were used to solve the final structure with molecular replacement methods and the structure of the truncated BlaC enzyme [10] (Table S3 in Tables S1). Despite the inclusion of the authentic N-terminal residues, the overall structure of full-length BlaC is very similar to the previously solved structure for truncated BlaC, a monomer containing an α domain and an α/β domain (Fig. S6).

**Figure 4:**
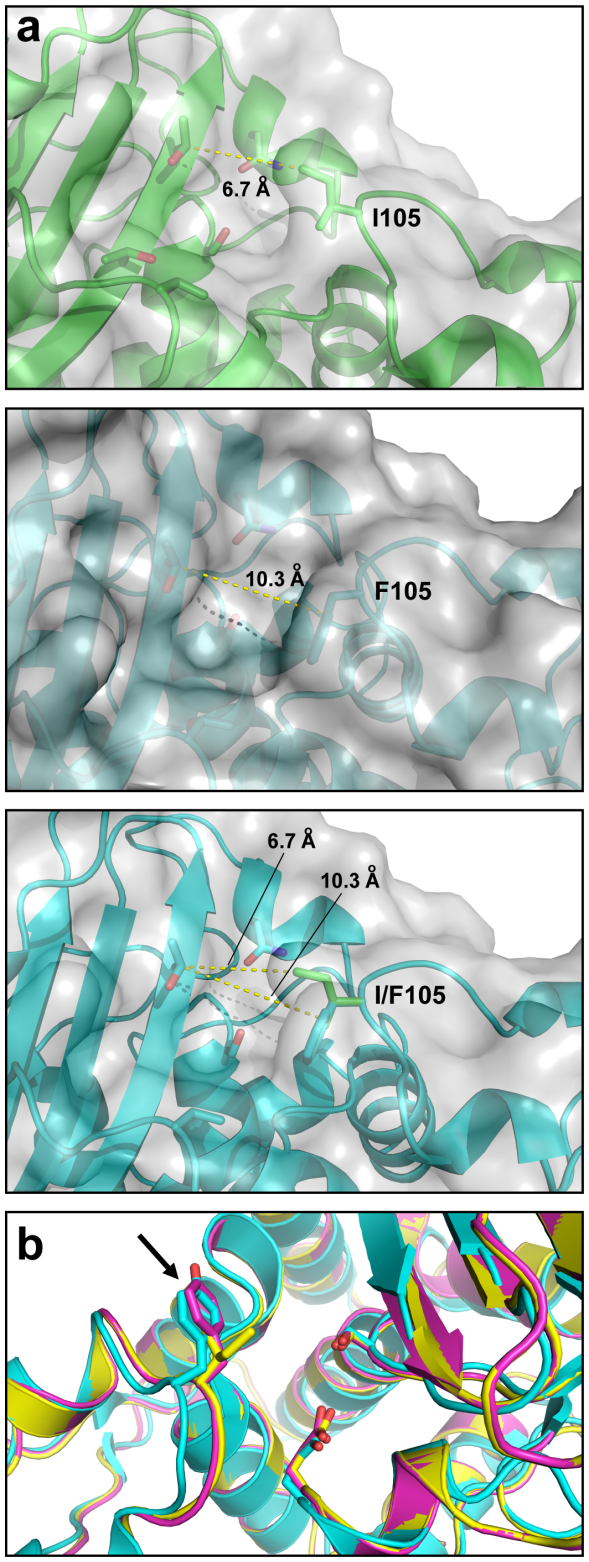
Structural basis for enhanced BlaC-mediated resistance. (a) Active sites of wt BlaC (top), BlaC(I105F) (middle), or structural alignment of both (bottom). (b) Structural alignment of wt BlaC (yellow), BlaC(I105F) (cyan), and TEM-1 Bla (magenta). Arrow indicates aromatic residues of BlaC(I105F), and TEM-1 Bla.

Previous sequence alignment of BlaC with other known class A β-lactamases revealed that the region ^102^IRSISP^107^ of *M. tuberculosis* BlaC, which harbors the I105F substitution, is quite different from other class A β-lactamases [10]. Whereas most other class A β-lactamases have either tyrosine or histidine with their aromatic side chains covering the entrance to the active site, Ile^105^ at the same position makes BlaC’s active site 3 Å wider than other class A β-lactamases [10]. Interestingly, our I105F substitution reintroduces an aromatic side chain in this position. While we were unable to solve the structure for BlaC(I105F), the structural impact of this substitution in the context of the neighboring BlaC residues was investigated by modeling the I105F substitution in the wt BlaC structure. Specifically, we found that the measured distance from Ile^105^ to a nearby active site residue was 6.7 Å whereas the distance from the modeled Phe^105^ mutation to the same nearby residue was 10.3 Å (Fig. 4a), signifying a significant increase in the opening of the mutant enzyme’s active site compared to the parental enzyme. Further structural alignment of the computationally modeled I105F mutant with both wt BlaC and TEM-1 Bla revealed that the mutant’s active site more closely resembles that of TEM-1 Bla, with the Phe^105^ mutation aligning closely with an active site Tyr residue of TEM-1 Bla (Fig. 4b). While this structural model is insufficient to unequivocally establish how this mutation improves catalytic efficiency, it suggests that Ile^105^ of BlaC may function as a ‘gatekeeper’ residue that regulates substrate accessibility to the enzyme active site. Remodeling of the BlaC active site by substitution with a Phe^105^ results in an enzyme that is more active towards β-lactams, which might be explained by the structural similarity between the mutant and TEM-1 active sites. Interestingly, protein kinases targeted by small-molecule inhibitors develop resistance through mutation of a gatekeeper threonine residue in the active site [27], reminiscent of the gain-of-function observed here when the putative gatekeeper residue of BlaC was mutated.

## Discussion

A key aspect of our studies was the functional transfer of BlaC-mediated drug resistance to *E. coli*, which enabled an evolutionary strategy for revealing mutational pathways that lead to enhanced antibiotic resistance associated with BlaC. After just a single round of mutagenesis and selection, we isolated variants of the BlaC enzyme that conferred significantly increased Amp resistance to *E. coli* and *M. smegmatis* cells compared to the same cells expressing wt BlaC. The *in vivo* effectiveness observed for BlaC(I105F) stems from measured improvements in a number of interrelated factors including catalytic efficiency, thermodynamic stability, and *in vivo* folding and transport efficiency that determines the concentration of active protein in the periplasmic space of the cell.

The antibiotic resistant phenotype was only observed when BlaC was fused to a Tat-specific leader peptide, ssTorA, confirming that BlaC is a Tat-dependent enzyme in *E. coli*. Consistent with previous studies in mycobacteria [12], BlaC is a dedicated Tat substrate in *E. coli* and could not be routed to the periplasm by the Sec translocase. Specifically, BlaC hybrids carrying Sec- and SRP-dependent export signals accumulated in the cytoplasm and cells expressing these constructs were susceptible to Amp. Given that BlaC does not incorporate a complex cofactor like many other Tat-dependent enzymes, a possible explanation for BlaC’s incompatibility with the Sec pathway may be that it folds too quickly to remain competent for export via the Sec translocase. Another possibility is that BlaC may be incapable of reaching a biologically active conformation in extracytoplasmic compartments and thus must fold in the cytoplasm prior to membrane translocation.

In contrast to ssTorA-BlaC, the full-length *M. tuberculosis* BlaC protein carrying its native signal peptide was not able to protect *E. coli* from β-lactam antibiotics. The full-length enzyme was expressed but not exported out of the cytoplasm by the *E. coli* Tat translocase. Therefore, the incompatibility that was previously observed between native BlaC and the *E. coli* Tat pathway [28] appears to derive from the mycobacterial signal peptide and not the mature enzyme. It is possible that the *M. tuberculosis* signal peptide is not recognized in *E. coli*, as the Tat machineries of *M. tuberculosis* and *E. coli* differ [29]. Another possibility is that an unidentified *M. tuberculosis* chaperone specific for the BlaC signal peptide may be required for productive export from the cytoplasm, as has been observed previously for other Tat substrates whose export depends on a dedicated chaperone [30]. *E. coli* may naturally lack this chaperone, which would explain why Tat export of full-length BlaC in *E. coli* fails to occur.

While ours is the first report to use directed evolution to uncover mutational pathways leading to enhanced BlaC-mediated resistance, others have previously applied similar strategies to the study of TEM-1 Bla [31]. By carefully controlling the *in vitro* evolution conditions (e.g., low mutagenesis rate, selection pressure similar to that seen by organisms in nature), directed evolution is able to accurately mimic natural evolution and can therefore be used to predict the results of natural antibiotic resistance [24]. Our use of a low mutation rate and moderate selection pressure for BlaC yielded mutants, in particular the single-substitution I105F allele, that may be predictive of future mutations that lead to increased antibiotic resistance. This mutant efficiently hydrolyzes β-lactams even in the face of mechanism-based inhibitors such as clavulanate, which is significant given the growing use of drug combinations in treating *M. tuberculosis* infections [11].

## Materials and Methods

### Bacterial Strains and Plasmids

Strain MC4100 and its isogenic derivative B1LK0 (Δ*tatC*) [21] were used for all experiments involving *E. coli*. *E. coli* ElectroMAX™ DH5α strain (Invitrogen) was used for plasmid DNA cloning and *E. coli* strain BL21(DE3) (Novagen) was used for expression and purification of BlaC enzymes. For susceptibility testing in mycobacteria, *M. smegmatis* strain PM965 (*ept-1 rpsL4* Δ*blaS1*) was used [7].

### Selective Plating of Bacteria

Bacterial plating was performed as described [16], [20]. Briefly, MC4100 or Δ*tatC* cells harboring one of the pSALect plasmids were grown overnight at 37°C in Luria Bertani (LB) medium supplemented with 25 µg/ml chloramphenicol (Cam). The next day, each culture was diluted to a density of 2.8×10^4^ cells/ml. 100 µL of normalized culture was removed, media-exchanged with fresh LB (no antibiotics), and subsequently serial diluted by factors of ten in a 96-well tissue culture plate. Aliquots of 5 µL from each well were spotted onto LB-agar plates containing Cam (control) or increasing Amp concentrations (0–600 µg/ml) and plates were incubated at 37°C for ∼16.5 h. To determine the effect of clavulante, similar experiments were performed using LB-agar plates supplemented with 0.025 or 0.25 µM clavulante and or increasing Amp concentrations (0–200 µg/ml). For antimicrobial susceptibility testing, zones of inhibition measured by the disk diffusion method with Sensi-discs (Becton Dickinson) were used to assay the antibiotic susceptibility of *M. smegmatis* strains as previously described [7].

### Plasmid Construction

For transfer of BlaC-mediated resistance to *E. coli*, the gene encoding full-length *M. tuberculosis* BlaC (Rv2068c), including its native Tat-dependent signal peptide, was PCR amplified and cloned into pSALect [16]. To express the BlaC protein with an *E. coli* signal peptide, the DNA encoding the mature region of BlaC was PCR-amplified and cloned between the *Nde*I and *Eco*RI sites of pSALect. The resulting plasmid, pSALect-ssTorA-BlaC-FLAG, expressed BlaC with an N-terminal ssTorA signal peptide and a C-terminal FLAG epitope tag. Derivatives of this plasmid were created by replacing the DNA encoding the ssTorA signal peptide with PCR-amplified DNA corresponding to the signal peptides of *E. coli* MBP, PhoA, and DsbA [32]. A version of BlaC lacking its signal peptide, ΔspBlaC, was created by PCR amplifying the mature domain of BlaC and cloning the resulting product into pSALect. To evaluate the resistance conferred by TEM-1 Bla, the plasmid pSALect-ssTorA-Bla was used [16]. For expression and purification studies, the genes encoding the wt and BlaC mutants were PCR-amplified and cloned between the *Nde*I and *Hin*dIII sites of pET-28b (Novagen). The resulting plasmids expressed wt and mutant BlaC enzymes with an N-terminal 6x-His tag followed immediately by a thrombin cleavage site. For expression of BlaC enzymes in mycobacteria, PCR-amplified DNA encoding the wt and mutant enzymes was cloned in plasmid pMV261 [33], resulting in plasmids pMP1070 and pMP1071, respectively. All plasmids generated in this study were verified by sequencing.

### Construction and Selection of Gene Libraries

For construction of BlaC enzyme library, mutant sequences were constructed using a modified procedure as described elsewhere [16], [20]. Briefly, the wt *blaC* gene was subjected to random mutagenesis by error-prone PCR using the GeneMorph II Mutagenesis Kit (Stratagene). The error-prone PCR conditions (initial amount of target DNA = 100 ng; number of PCR cycles = 25) were chosen to favor low mutation frequencies (0–4.5 mutations/kb). The resulting PCR products were cloned into the *Nde*I and *Eco*RI sites of pSALect-ssTorA-BlaC-FLAG in place of wt *blaC*. The ligation products were transformed into ElectroMAX DH5α cells resulting in a library of ∼1×10^6^ independent transformants. Sequencing of ten clones selected at random revealed an average mutation rate of two nucleotides per gene. The *blaC* gene library was midiprepped from DH5α and used to transform electrocompetent MC4100 cells. Transformed cells were plated on LB-agar containing Cam and 0.2% glucose and the next day, colonies were counted to ensure the diversity of the library was maintained. Library cells were pooled and grown overnight in LB medium supplemented with Cam and 0.2% glucose. To select positive clones, overnight cells were serially diluted, plated on Amp (200 µg/ml), and incubated overnight at 30°C. Stable clones were yielded by counter selection on LB-agar plates with equal amounts of Amp (200 µg/ml). Plasmids isolated from stable clones were back-transformed into MC4100 cells, and the resulting transformants were re-tested for growth on Amp. Purified plasmids from single selected clones were sequenced and mutants that reproducibly conferred the greatest Amp resistance were obtained and studied further.

### Subcellular Fractionation and Western Blot Analysis

Overnight cells were subcultured ten-fold in LB containing antibiotics and allowed to grow for an additional 1.5 h at 37°C until a cell density (*A*_600_) of ∼0.5 was reached, at which time the cultures were induced with 1 mM IPTG and incubated at 30°C. Protein expression proceeded at 30°C for ∼16.5 h, after which cultures were normalized by *A*_600_ and culture aliquots were pelleted via centrifugation for 15 min at 4°C and 3,500 rpm. For preparation of cell lysates, pellets were resuspended in phosphate buffered saline (PBS) and lysed by addition of BugBuster (Novagen). For subcellular analysis, cell pellets were subsequently fractionated according to the ice-cold osmotic shock method as described elsewhere [13]. Proteins were separated by SDS-PAGE using 12% polyacrylamide gels (Bio-Rad) and subsequently detected by Western blotting according to standard protocols using the following primary antibodies: mouse anti-FLAG (Abcam) and rabbit anti-GroEL (Abcam) [34].

### Protein Purification

All BlaC enzymes were expressed and purified from BL21(DE3) carrying pET-28b plasmids encoding the wt or mutant BlaC enzymes. Cells were grown in TB broth supplemented with 100 µg/ml Amp until mid-log phase, at which time gene expression was induced by addition of 0.5 mM IPTG followed by growth for an additional 10 h at 20°C and shaking at 150 rpm. Induced cells were harvested and resuspended in 150 mM Na_2_HPO_4_ pH 8.0, 300 mM NaCl supplemented with 100 µM PMSF and disrupted with a microfluidizer (Microfluidics). The cleared lysate (150,000×*g*, 40 min, Ti-45) was applied to Ni-NTA affinity resin (GE Healthcare) and protein was eluted with an imidazole gradient using an ÄKTA FPLC system (GE Healthcare). BlaC-containing fractions were pooled and dialyzed against 50 mM Tris pH 8.0, 150 mM NaCl, and thrombin was added to cleave the N-terminal 6x-His tag. Size exclusion chromatograhpy was performed on a Superdex 200 26–60 HighLoad column (GE Healthcare) using the same buffer.

### Enzyme Activity Assays

BlaC activity in cell lysates was determined by adding 1 µl of cleared lysate to 10 µl of 10×PBS and 39 µl ddH2O. The reaction was initiated by the addition of 50 µM nitrocefin and monitored at 486 nm for 10 min. Activity of the purified enzymes was measured in steady state by monitoring the hydrolysis of ampicillin and nitrocefin exactly as described previously [10] using a Beckman DU Spectrophotometer at 25°C. Initial velocities were fitted to the equation below using GraFit.
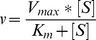
where *v* is the initial velocity, *V*_max_ is the maximal velocity, and *K_m_* is the Michaelis constant for the substrate, S. Data was fitted to the following equations, determining *K_m_* and *k*_cat_.



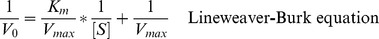


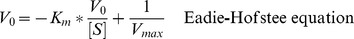


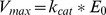



*E*_0_ =  initial enzyme concentration;

*k*_cat_ = turnover number.

### Urea Induced Unfolding

The equilibrium unfolding transitions of BlaC enzymes were measured using a Jasco FP6500 fluorescence spectrophotometer in 100 mM potassium phosphate, pH 7.4, 0–6 M urea at 25°C. Each protein (0.5 µM) was incubated in different concentrations of urea for 1 h at 25°C. The fluorescence of each sample was recorded at 330 nm (5 nm band width) in a 1-cm cuvette after excitation at 280 nm (3 nm band width). The concentration of urea in each sample was calculated from the refractive index. The data were analyzed according to a two-state model of unfolding [35] using GraFit (Erithacus software). The Gibb’s free energy of unfolding Δ*G* and the cooperativity parameter *m* were used to calculate the midpoint of the transition [urea]_M_ [36].

### Enzyme Inhibition Assays

Clavulante was used in concentrations ranging from 0 to 70 µM in the presence of 100 µM nitrocefin to determine IC_50_ values in 100 mM MES pH 6.4. Reaction was initiated by addition of 2 nM of wt BlaC or mutant enzyme. Absorption at 486 nm (ε = 20,500 M^−1^cm^−1^) was monitored for 10 min at 25°C. Initial velocities were fitted the following equation and IC_50_ determined.
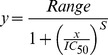
where *Range* = fitted uninhibited value (clavulanate 0 µM) and *s* = slope factor.

### Protein Crystallization

Initial crystallization conditions were determined at 20°C using commercial screens (Qiagen). Concentrated protein (20, 25 and 30 mg/ml) was mixed 1∶1 with buffer in a volume of 0.1 µl : 0.1 µl, using sitting drop method. Hanging drop plates were used to optimize initial hits. Protein solution (18 mg/ml) and mother liquor containing 2.1 M NH_4_SO_4_ and 100 mM TRIS/HCl pH 7.8 was mixed in equal ratio. Plates were incubated at 20°C. After three days first crystals were observed. Several rounds of macroseeding improved crystal shape and diffraction.

### Data Collection

Prior to data collection, crystals were cryo-protected with mother liquor supplemented with 20% PEG-400, then flash-frozen in liquid nitrogen. Diffraction data was collected at Swiss Light Source on beamline PXII - X10SA. Data was processed with XDS [37]. Molecular replacement was done with Molrep [38] from the CCP4 suite [39] using an existing model (pdb accession code 2GDN). Density improvement and refinement was carried out with *PHENIX* [40].

## Supporting Information

Figure S1 (PDF)

Figure S2 (PDF)

Figure S3 (PDF)

Figure S4 (PDF)

Figure S5 (PDF)

Figure S6 (PDF)

Tables S1 **Includes Table S1, S2, and S3.**
(PDF)
