# Supplementary material for: Directed Evolution of Mycobacterium tuberculosis β-Lactamase Reveals Gatekeeper Residue That Regulates Antibiotic Resistance and Catalytic Efficiency
Source: PLoS One. 2013 Sep 4;8(9):e73123. doi: 10.1371/journal.pone.0073123 (PMC3762836; doi:10.1371/journal.pone.0073123)
Supplement: Figure S1 — (PDF) [file pone.0073123.s001.pdf]

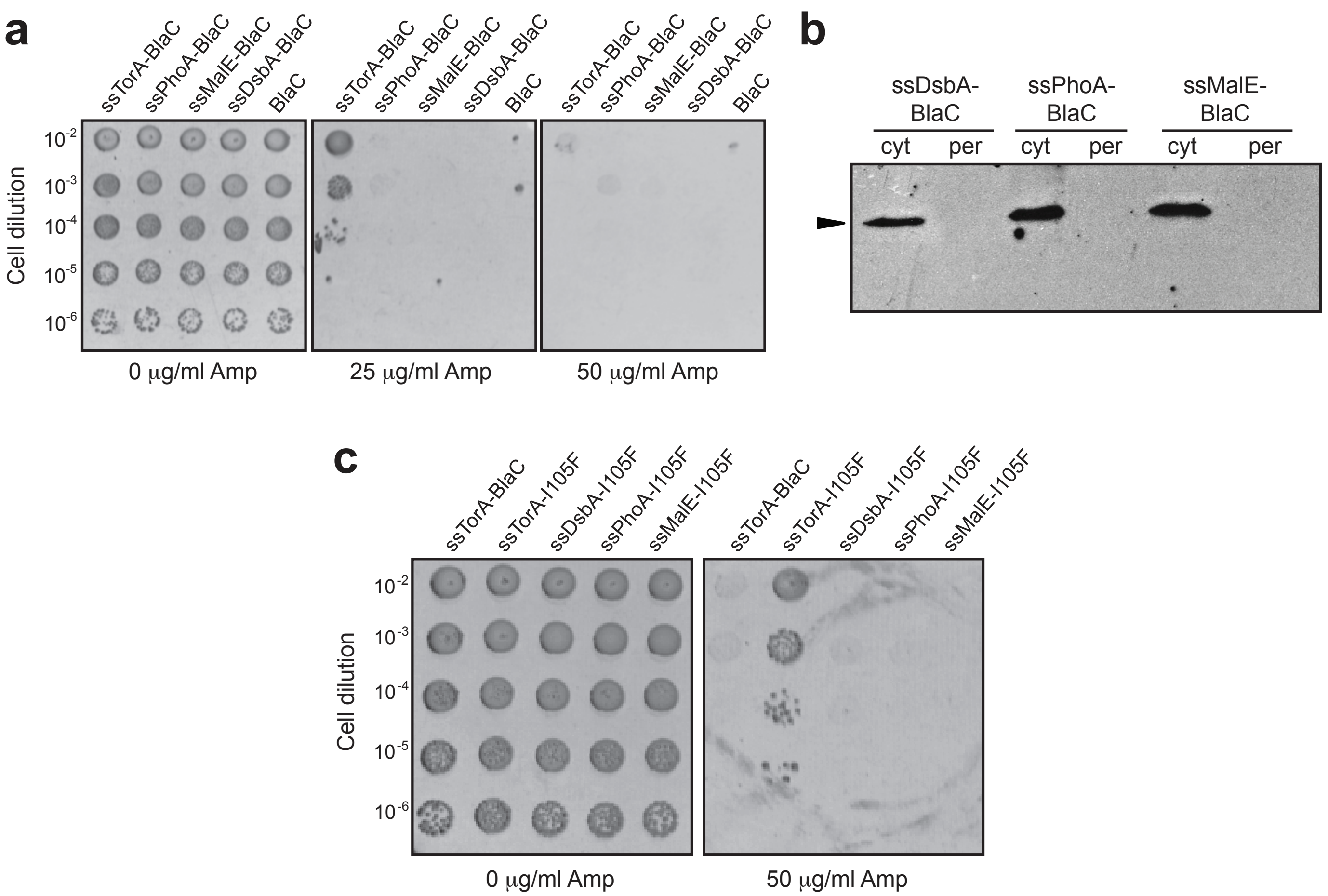

Supplemental Figure S1. Evaluation of antibiotic resistance in *E. coli*. (a) Serially diluted wt or Δ*tatC* cells expressing ssTorA-BlaC, full-length BlaC, or BlaC hybrids with Sec- and SRP-dependent export signals. (b) Western blot analysis of cytoplasmic (cyt) and periplasmic (per) fractions prepared from wt cells expressing BlaC hybrids with Sec- and SRP-dependent export signals. Arrow indicates BlaC. Samples prepared from an equivalent number of cells were loaded in each lane. Blots were probed with anti-FLAG antibodies. (c) Serially diluted wt cells expressing ssTorA-BlaC, ssTorA-BlaC(I105F), or BlaC(I105F) hybrids with Sec- and SRP-dependent export signals were spotted on Amp.
