# Supplementary material for: Directed Evolution of Mycobacterium tuberculosis β-Lactamase Reveals Gatekeeper Residue That Regulates Antibiotic Resistance and Catalytic Efficiency
Source: PLoS One. 2013 Sep 4;8(9):e73123. doi: 10.1371/journal.pone.0073123 (PMC3762836; doi:10.1371/journal.pone.0073123)
Supplement: Figure S2 — (PDF) [file pone.0073123.s002.pdf]

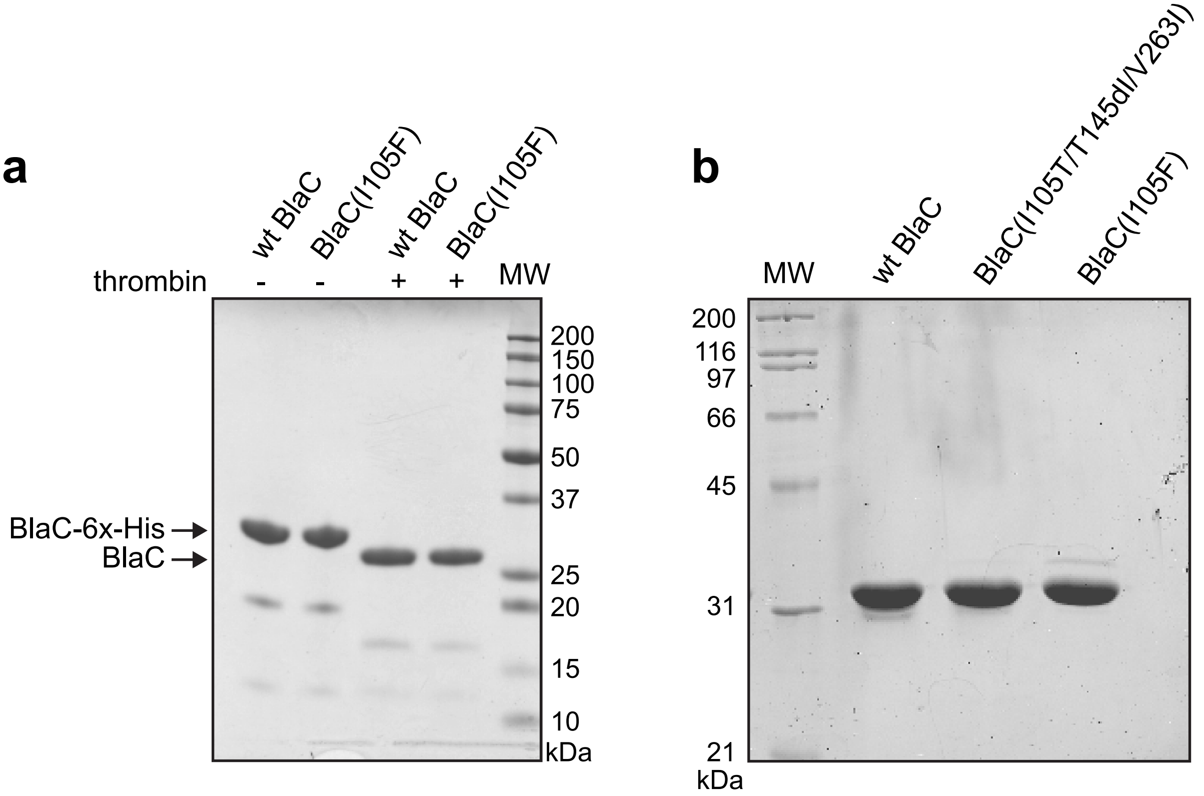

Supplemental Figure S2. Purification of wt and mutant BlaC enzymes. The wt and BlaC mutant enzymes were all purified by Ni-NTA chromatography followed by size exclusion chromatography. An equal amount of each purified enzyme was loaded onto an SDS-PAGE gel, which was subsequently stained with Coomassie blue to visualize proteins. (a) Following purification, the N-terminal 6x-His tag was removed by thrombin cleavage. (b) For nitrocefin analysis, the BlaC(I105T/T145dI/V263I) mutant was purified in addition to wt and BlaC(I105F) mutants. MW, molecular weight ladder. The expected size of BlaC with 6x-His tag is ~32 kDa.
