# Supplementary material for: Directed Evolution of Mycobacterium tuberculosis β-Lactamase Reveals Gatekeeper Residue That Regulates Antibiotic Resistance and Catalytic Efficiency
Source: PLoS One. 2013 Sep 4;8(9):e73123. doi: 10.1371/journal.pone.0073123 (PMC3762836; doi:10.1371/journal.pone.0073123)
Supplement: Figure S3 — (PDF) [file pone.0073123.s003.pdf]

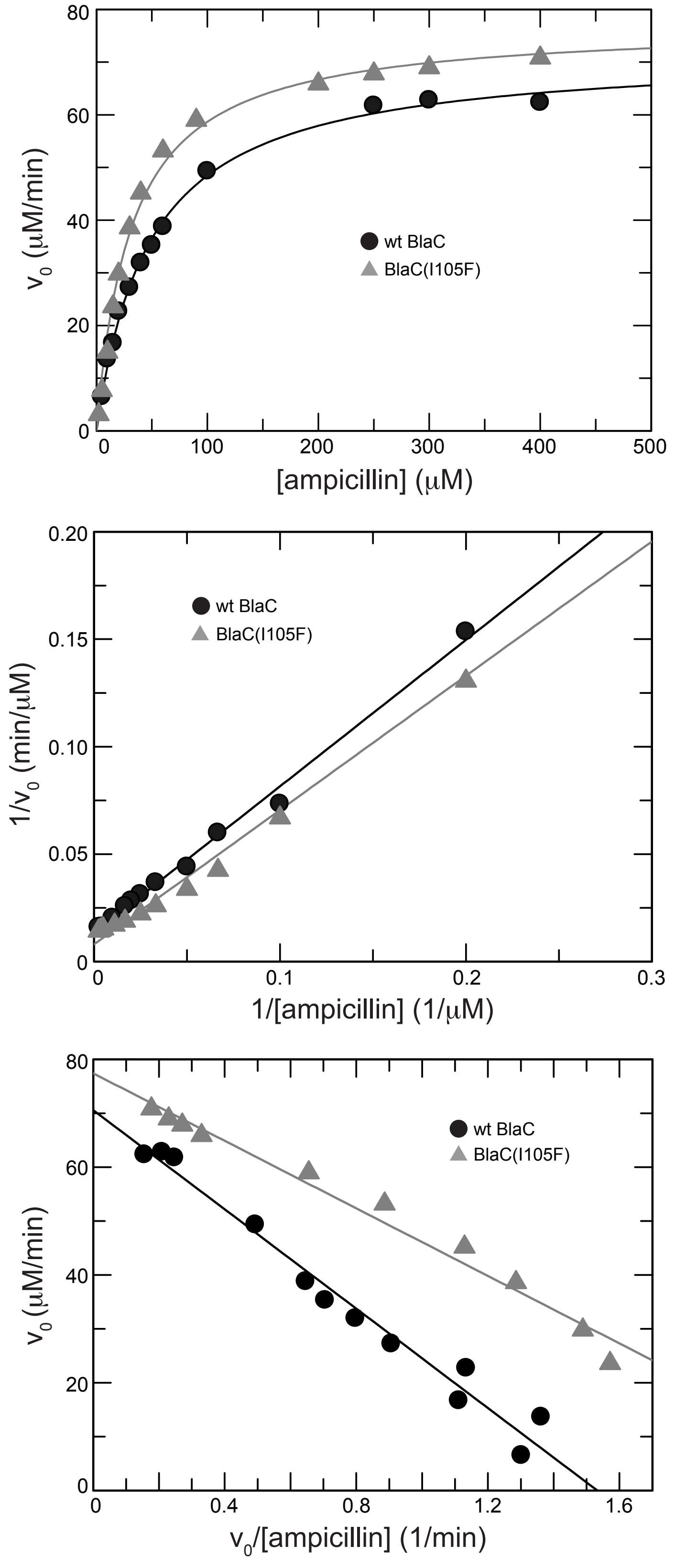

Supplemental Figure S3. Kinetics of wt and mutant BlaC enzymes. (a) Michaelis-Menten kinetics (top), Lineweaver-Burk plot (middle), and Eadie-Hofstee plot (bottom) generated for wt BlaC and BlaC(I105F) enzymes with Amp at single enzyme concentration (50 μM).
