# Supplementary material for: Directed Evolution of Mycobacterium tuberculosis β-Lactamase Reveals Gatekeeper Residue That Regulates Antibiotic Resistance and Catalytic Efficiency
Source: PLoS One. 2013 Sep 4;8(9):e73123. doi: 10.1371/journal.pone.0073123 (PMC3762836; doi:10.1371/journal.pone.0073123)
Supplement: Figure S5 — (PDF) [file pone.0073123.s005.pdf]

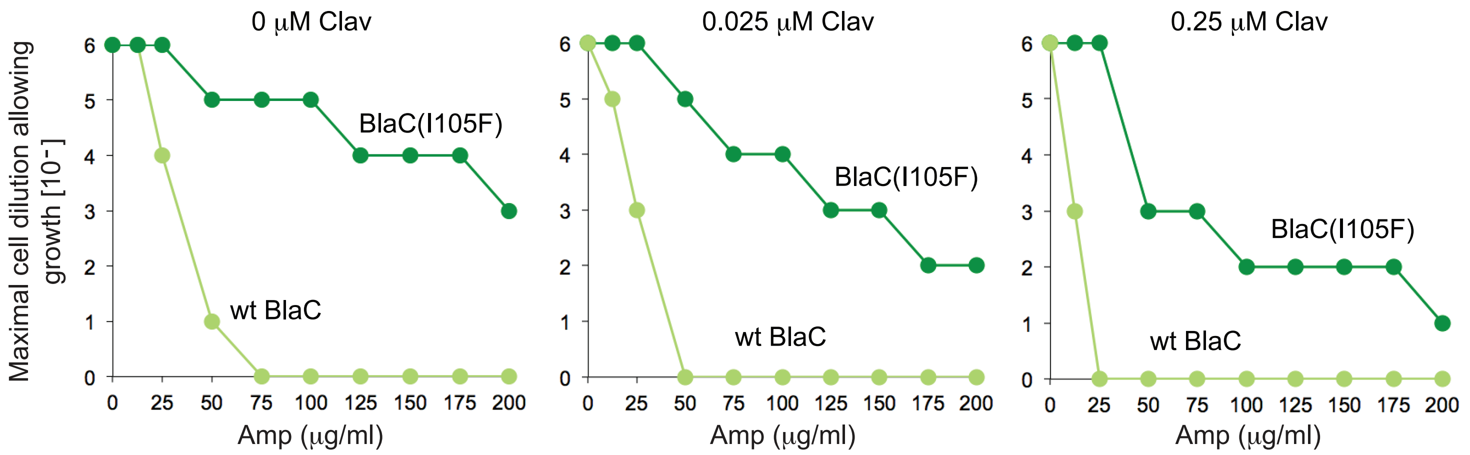

Supplemental Figure S5. Bacterial susceptibility to Amp-Clav combination. Amp resistance of wt cells expressing ssTorA-BlaC and ssTorA-BlaC(I105F) in the presence and absence of the inhibitor clavulanate as indicated. Maximal cell dilution that allowed growth is plotted versus Amp concentration.
