# Supplementary material for: Directed Evolution of Mycobacterium tuberculosis β-Lactamase Reveals Gatekeeper Residue That Regulates Antibiotic Resistance and Catalytic Efficiency
Source: PLoS One. 2013 Sep 4;8(9):e73123. doi: 10.1371/journal.pone.0073123 (PMC3762836; doi:10.1371/journal.pone.0073123)
Supplement: Figure S6 — (PDF) [file pone.0073123.s006.pdf]

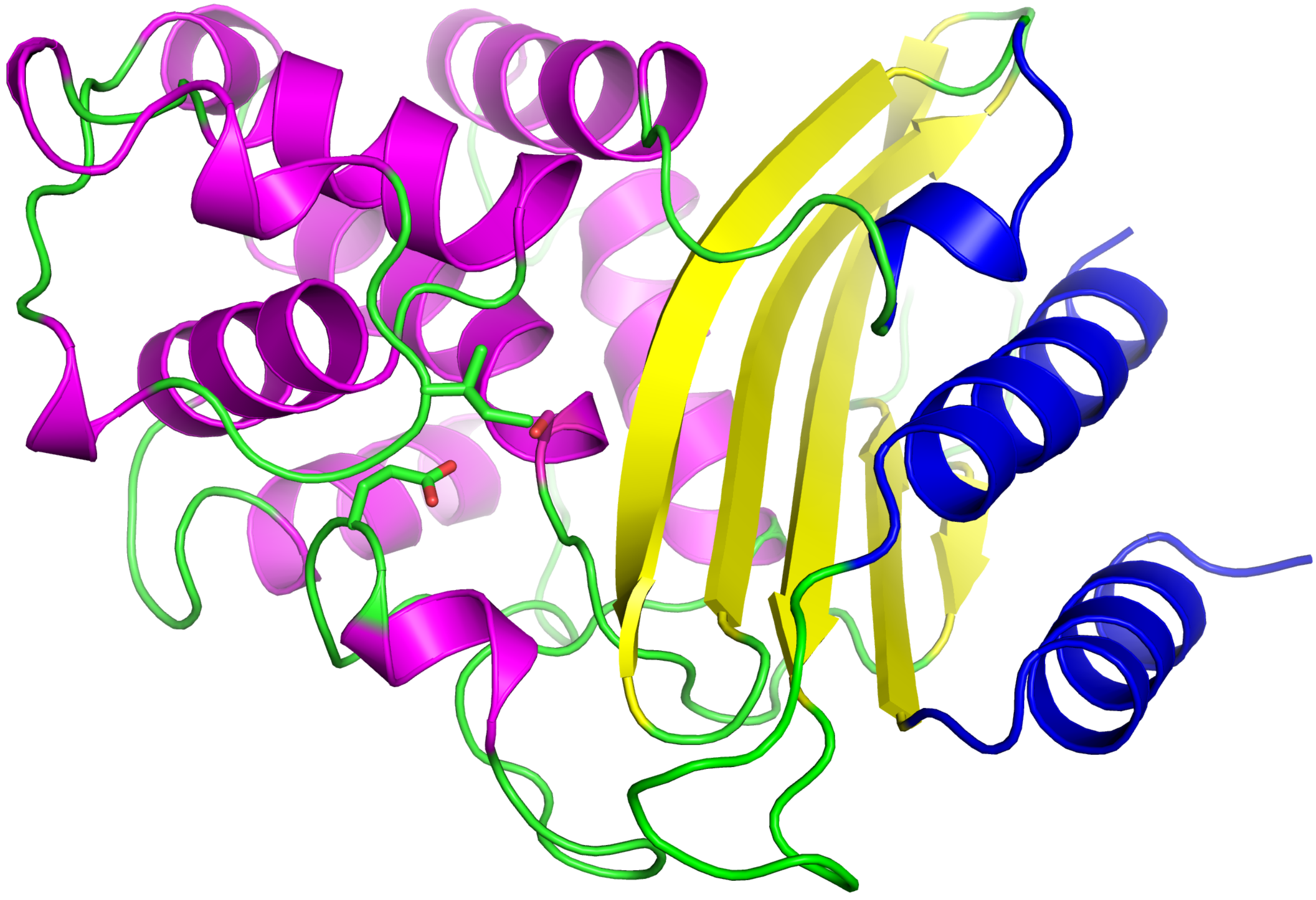

Supplemental Figure S6. Overall structure of full-length *M. tuberculosis* BlaC and its secondary structures. Clearly visible are the α domain and an α/β domain.
