# Supplementary material for: Directed Evolution of Mycobacterium tuberculosis β-Lactamase Reveals Gatekeeper Residue That Regulates Antibiotic Resistance and Catalytic Efficiency
Source: PLoS One. 2013 Sep 4;8(9):e73123. doi: 10.1371/journal.pone.0073123 (PMC3762836; doi:10.1371/journal.pone.0073123)
Supplement: Tables S1 — Includes Table S1, S2, and S3. (PDF) [file pone.0073123.s007.pdf]

Supplemental Table S1. Additional kinetic parameters for BlaC enzymes

Enzyme	V_{max} (µM/min)[a]	K_m (µM)[a]	k_{cat} (min^{-1})[a]	k_{cat}/K_m (min^{-1} M^{-1})[a]
wt BlaC	12.1 ± 0.4	45 ± 4	2421 ± 88	(5.4 ± 0.3) x 10^7
BlaC(I105T/T145dI/V263I)	12.3 ± 0.2	36 ± 1	2468 ± 38	(7.1 ± 0.2) x 10^7
BlaC(I105F)	11.9 ± 0.4	16 ± 2	2380 ± 80	(1.5 ± 0.1) x 10^8

[a]Kinetic parameters determined using nitrocefin as substrate

Supplemental Table S2. Antibiotic susceptibilities of *M. smegmatis* expressing BlaC enzymes

Strain	Genotype	Disk diffusion diameter (mm)[1]					
		Amp^{500}	Amp^{50}	Amp^{50} $Clav^{0.50}$	Amp^{50} $Clav^{0.25}$	Amp^{50} $Clav^{0.125}$	Amp^{50} $Clav^{0.06}$
PM965/pMV261	(Δ*blaS1*/vector control)	47 ± 1	29 ± 2	-	-	-	-
PM965/pMP1070	(Δ*blaS1*/*blaC*+)	30 ± 1	15 ± 2	20 ± 1	19 ± 2	18 ± 2	15 ± 1
PM965/pMP1071	(Δ*blaS1*/*blaC*-I105F+)	20 ± 2	0 ± 1	19 ± 1	18 ± 1	15 ± 1	11 ± 1

[1]For disk diffusion tests, the zones of inhibition are averages ± standard deviations of duplicate determinations for two independent cultures of each strain. Amp was used at a concentration of 500 or 50 µg/disk as indicated and Clav was in the agar media at 0.06-0.50 µg/mL as indicated.

Supplemental Table S3. Data collection, processing, and refinement statistics

Parameter	Value for wt BlaC[1]
Wavelength (Å)	1.000
Resolution range (Å)	19.87 - 2.85 (2.95 - 2.85)
Space group	$P2_1$
Unit cell	a = 78.8 b = 96.5 c = 111.0<br>α = γ = 90 β = 108.5
Total reflections	116897 (11344)
Unique reflections	36698 (3607)
Multiplicity	3.2 (3.2)
Completeness (%)	99.7 (99.7)
Mean I/σ(I)	12.52 (4.07)
R-factor	0.17 (0.24)
R-free	0.22 (0.30)
Number of atoms	15936
Macromolecules	7947
Ligands	85
Water	102
Protein residues	1064
RMS(bonds)	0.015
RMS(angles)	1.44
Ramachandran favored (%)	97
Ramachandran outliers (%)	0
Clashscore	9.73
Average B-factor	9.80
Macromolecules	9.40
Solvent	7.10
PDB accession code	3zhh

[1]Statistics for the highest resolution shell are shown in parentheses.
